# Commentary: Demystifying Doppler – revisiting a vital diagnostic tool

**DOI:** 10.1186/s13047-022-00530-x

**Published:** 2022-03-26

**Authors:** Peta Tehan, Jill Sommerset, Richard Rounsley, Martin Fox

**Affiliations:** 1 School of Clinical Sciences, Faculty of Medicine, Nursing and Health Sciences, Monash University, Clayton, Victoria Australia; 2 School of Health Sciences, College of Health, Medicine and Wellbeing, University of Newcastle, Ourimbah, NSW Australia; 3 PeaceHealth Southwest Medical Center, Washington, USA; 4 Vascular Healthcare, Lake Macquarie, NSW Australia; 5 Manchester Leg Circulation Service, Manchester Local Care Organisation(NHS), Harpurhey Health Centre, Manchester, UK

## Abstract

Doppler is the most commonly utilised vascular assessment tool by podiatrists in Australia and the United Kingdom. Doppler is a key component of many international guidelines for vascular assessment. Used alongside pressure measurements such as ankle and toe-brachial indices, Doppler assists podiatrists to diagnose, triage and subsequently manage patients with peripheral arterial disease. This commentary aims to clarify the importance, technique, and interpretation of continuous wave handheld Doppler in podiatry practice. This commentary presents discussion on the equipment and optimal test conditions for use of Doppler, and guidance on the technique required in podiatry clinical practice. Furthermore, there is a focus on interpretation of the output from Doppler including both audio and visual output. There is in depth discussion about identifying pathology and integration into the clinical management plan.

## Background

Following a recent vascular workshop at the Australian national podiatry conference, most delegates questions related to the use and interpretation of continuous wave Doppler. There is a desire for more information about the most commonly utilised vascular assessment tool by podiatrists in Australia, New Zealand, and the United Kingdom [1, 2]. Used alongside ankle and toe brachial indices, Doppler assists podiatrist to diagnose, triage, and subsequently manage patients with peripheral arterial disease (PAD) [3, 4]. Doppler has high levels of diagnostic accuracy, particularly in notoriously difficult to diagnose populations, such as diabetes and foot ulceration [3, 5]. Doppler has also been recommended as a key component of the initial lower limb vascular assessment in the Global Vascular Guidelines [6].

However, the reliability, definitions, descriptors, and associated interpretation of Doppler remain variable in the literature [7–9]. This commentary aims to clarify the importance, technique, and interpretation of continuous wave handheld Doppler in podiatry practice.

### Equipment

There are multiple brands and types of continuous wave handheld Doppler units available. The most appropriate Doppler for podiatrists will have an 8Mhz probe with a unit which has an audio and visual output. This is adequate for the depth of the vessels commonly insonated by podiatrists [10]. Ideally the unit should be compatible with photoplethysmography to enable measurement of toe systolic pressures. An additional 5Mhz probe can help with locating and interpreting deeper pulses, e.g. popliteal, femoral or if excess oedema is present.

### Test conditions

As with other vascular tests, ideally the patient should be in a supine, rested position prior to measurement [9, 10]. The foot should be relaxed, that is, not dorsiflexed or actively plantarflexed, as these positions can obliterate the signal [10]. The temperature of the room should be controlled between 20-25°C to avoid vasoconstriction of peripheral vessels [9].

### Technique and position

**Fig 1:**
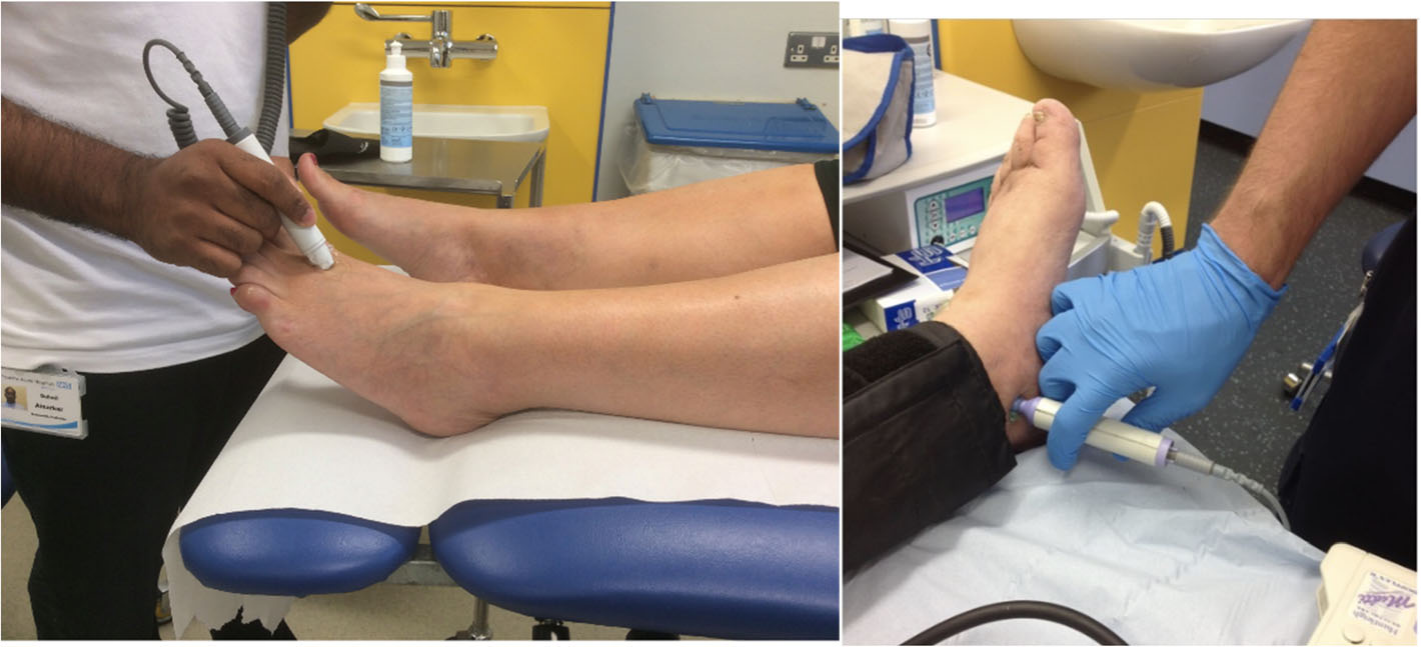
Image depicting anchored hand positioning for using Doppler on dorsalis pedis artery (L) and posterior tibial artery (R)

**Fig 2:**
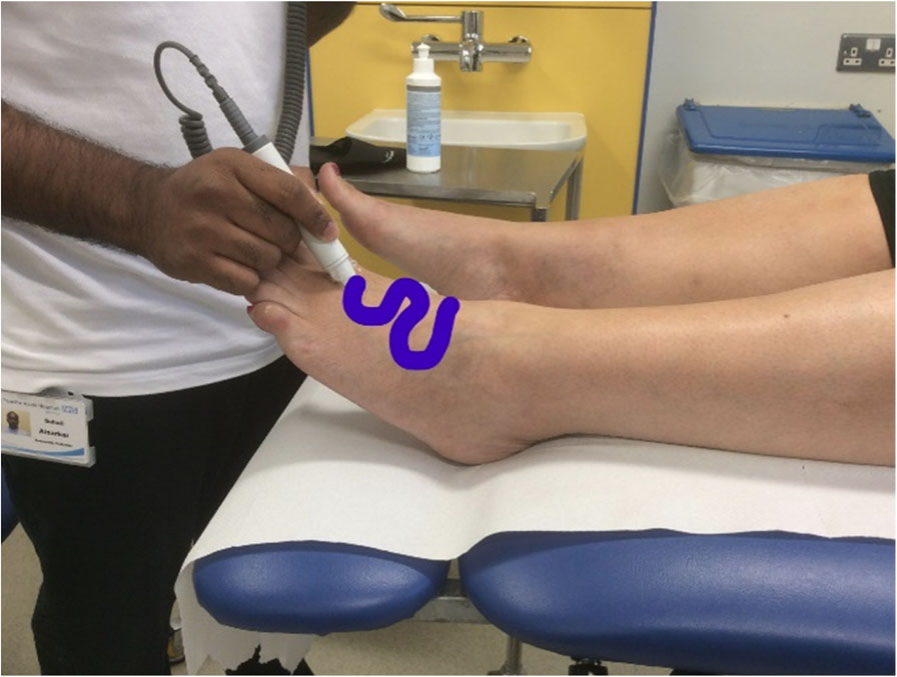
Image depicting S shape pattern which can be used on dorsum of foot to locate the dorsalis pedis pulse

Generally in the lower limb, the Doppler probe is pointed up the limb towards the head of the patient (cephalad) and angled at approximately 45° [11]. Adequate water-based coupling gel should be used to conduct the signal [11]. Care should be taken with the amount of pressure applied, as too much pressure will obliterate the signal or lead to an artificially resistant looking waveform [10]. Handling of the probe is generally using pencil grip, anchoring the hand to the limb or foot (Fig 1). This is particularly helpful when taking pressures to avoid the probe from moving when inflating and deflating the cuff, which would result in an inaccurate result. When searching for a waveform, an S or snake shape can be used, slowly tracing the probe across the dorsum of the foot to identify the artery (Fig 2). Once a signal is detected, smaller motions to move the probe from side to side a few degrees either way (fanning) or varying the angle, can be used to optimise the signal.

**Fig 3:**
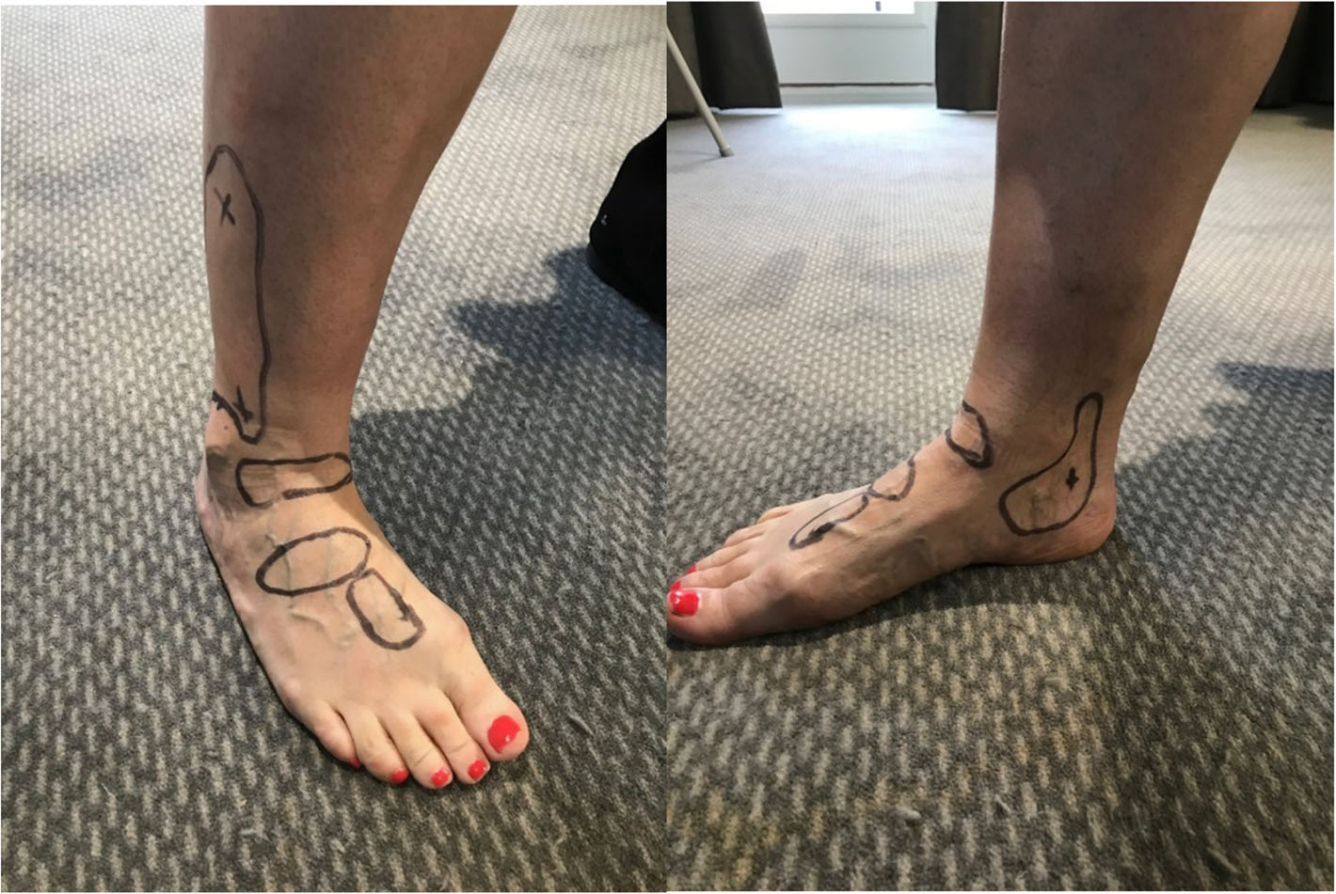
Locations for pulse insonation, Left) from proximal to distal, the peroneal pulse, anterior tibial, dorsalis pedis pulse, and the distal dorsalis pedis pulse Right) posterior tibial pulse

Two Doppler waveforms can be obtained in the foot, the posterior tibial and dorsalis pedis arteries, with the distal peroneal artery able to be detected in the distal leg, behind the lateral malleolus [10] (Fig 3), and the anterior tibial artery can be located at the anterior aspect of the ankle at the distal medial border of the tibia [10]. The posterior tibial artery can be insonated just posterior to the medial malleolus (Fig 3). There is very little anatomical variation of the posterior tibial artery at the level of the medial malleolus, as it lies within the tarsal tunnel in 91% of the population [12]. The posterior tibial artery and dorsalis pedis artery are similarly small calibre arteries (between 1.96-2.05 mm) [13] which can make detection challenging, with further difficulties arising in larger limbs, the presence of oedema and excess adiposity [11].

The dorsalis pedis artery, a continuation of the anterior tibial artery, has more anatomical variation to consider [14]. In the majority of cases the dorsalis pedis lies on the dorsum of the midfoot [10], between the extensor hallucis longus and extensor digitorum longus tendons, superior to the cuneiforms. The dorsalis pedis artery is congenitally absent in approximately 7% of the population [14], and has five different, typical anatomical patterns [15, 16]. This, combined with the small calibre of the artery [13] can make it more challenging to insonate. The anterior tibial artery, can be easier to locate at the distal medial border of the tibia [10], and clinicians may choose to trace the artery from this point, distally, to locate the dorsalis pedis signal. The peroneal artery can be insonated at the lateral lower leg, usually superior (above) to the lateral malleolus, with the probe pointed medially towards the tibia [10, 17] (Fig 3). It can sometimes be difficult to locate due to its small size, location and depth [11]. Current national guidelines for vascular assessment (United Kingdom) recommend the inclusion of foot, popliteal and femoral pulses in the lower limb vascular examination [18]. Inclusion of these additional pulses can be important, to help identify anatomical location and severity of arterial disease [10], as well as supporting diagnosis, triage and vascular referral decisions.

### Interpretation: audio and visual

**Fig 4:**
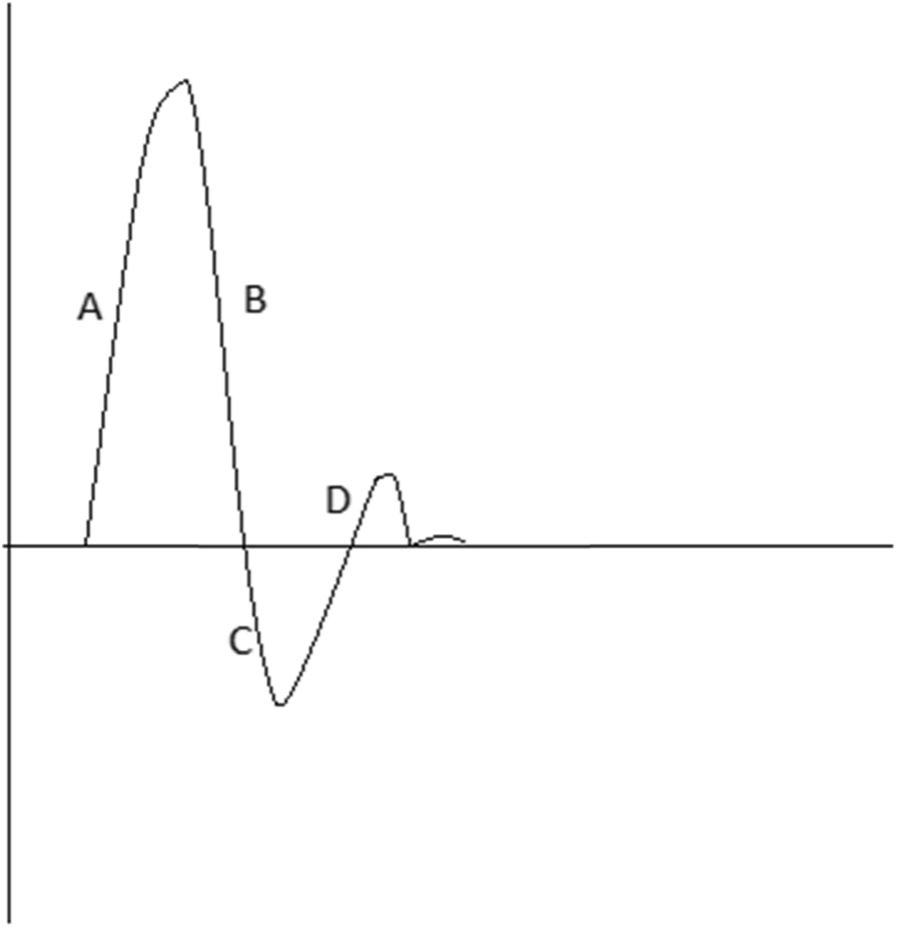
Anatomy of a Doppler waveform. **A** Systolic rise or upstroke, **B** Systolic downstroke, A+B=systole, **C** Early diastole, **D** Late diastole, horizontal axis= zero flow baseline

Traditionally in podiatry, waveforms are classified simplistically, as monophasic (one sound, forward flow only), biphasic (two sounds, forward and reverse flow), and triphasic (three sounds, forward, reverse flow and elastic recoil, often with a characteristic whip sound) (Fig 4) [19, 20]. Irregularity may be noted, and various other descriptors are often used to complement phase interpretation [17].

The interpretation of Doppler output remains qualitative, as opposed to pressure measurements which yield more quantitative data [8]. Doppler audio and visual outputs can be difficult or confusing to interpret, especially if they do not appear to agree [17]. For example, an audio can sound bi / tri phasic, when a visual output may identify it as monophasic, with intermediate or low resistance. For clinicians with audio-only Dopplers, this may affect decisions to further assess the vascular status, at worst resulting in a delay in diagnosis of arterial disease, in someone with a monophasic waveform, misinterpreted as bi/triphasic or ‘normal’. Any doubts about interpretation and subsequent clinical diagnostic decisions may be best addressed by using audio and visual waveform outputs together where possible, and adding pressure measurements and/or indices, such as ankle/toe pressures or ankle-brachial or toe-brachial indices to the clinical assessment [3, 17]. Pressure measurements and indices make a valuable addition to the clinical decision-making process, which is why a combination of testing methods while considering the clinical presentation is recommended [21].

### Ideal visual waveforms

Characteristics of an ideal visual waveform should include three phases, with sharp systolic upstroke (forward up rise and fall) and early diastolic flow reversal with a final forward flow during late diastole (Fig 4) [10, 22]. Biphasic waveforms are most commonly described as those which have lost their final forward flow (late diastole) [17], and can sometimes can be considered pathological [23], depending on their sounds and visual appearance. Nomenclature with standard definitions and descriptors have only recently reached consensus and proposed for use by all clinicians who utilise Doppler as part of their lower limb assessment [17]. The consensus document suggests for visual interpretation of Doppler waveforms, three major descriptors should be used, direction of flow (antegrade or retrograde), phasity (multiphasic or monophasic) and resistance (high, intermediate, or low) [17]. This may go some way to supporting podiatrists and other clinicians to standardise clinical education and training around interpretation and clinical diagnosis.

### Pathological waveforms

**Fig 5:**
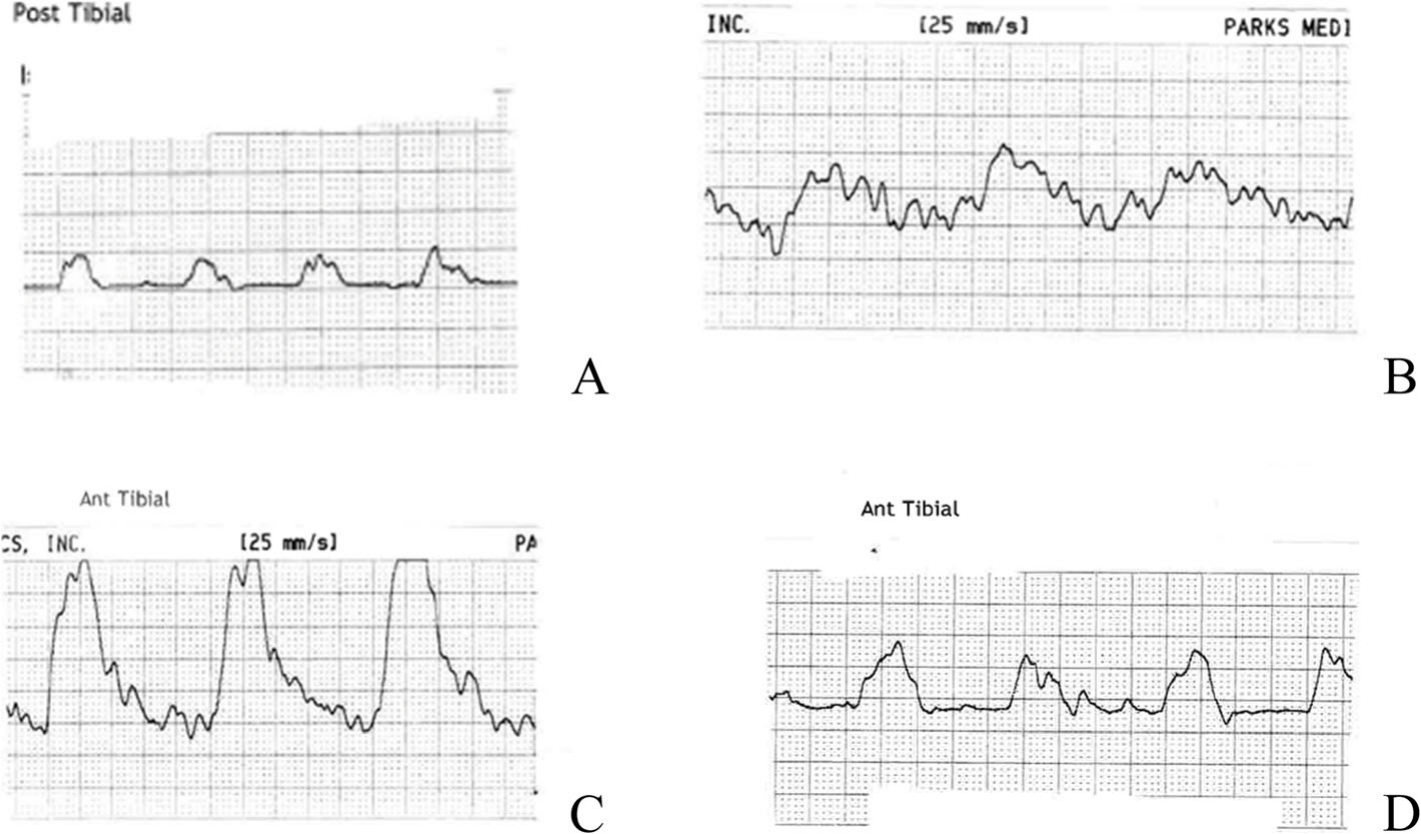
Examples of monophasic waveforms including decreased amplitude (**A**), dampening and turbulence (**B**), turbulence, (**C**), and attenuated with decreased amplitude (**D**)

It is important to understand how waveforms change in the presence of pathology, in order to accurately detect disease. Monophasic, or demodulated waveforms are generally considered indicative of PAD [3, 9], with a single phase and delayed systolic acceleration and absent diastole [10] (Fig 5). There is variation in the presentation of monophasic waveforms, depending on the severity and location of disease [17]. Changes include extended systolic rise time (attenuated), where the systolic upstroke is less vertical and can result in a wider looking systolic complex, reduction in the overall size of waveform (decreased amplitude), and reduction in maximum systolic velocity and extended systolic rise time (dampening/blunting) which appears as a smaller and broader systolic complex [9, 10, 22]. Turbulence, which presents as serrated contours on a widened systolic peak, should also be considered pathological [10] as it represents non-linear flow as a result of a proximal obstruction.

**Fig 6:**
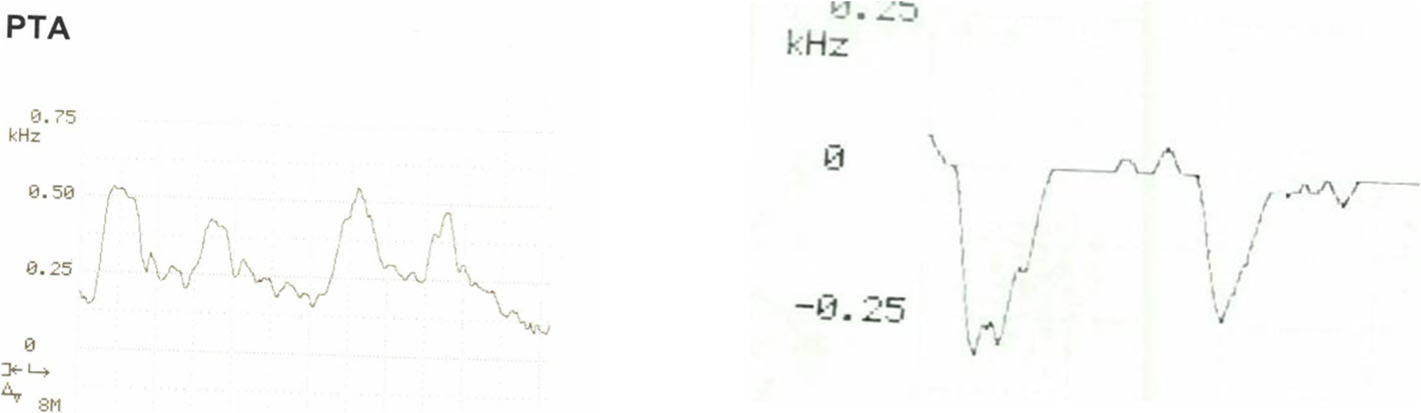
(left) hyperaemic waveform – waveform sits above the zero flow baseline (horizontal axis), and (right) retrograde waveform – waveform appears to be upside down

Changes in peripheral resistance, which occur in the presence of inflammation, infection or post exercise, can also lead to changes in the appearance of waveforms [24]. This leads to waveforms appearing above the horizontal axis (zero baseline) [10] (Fig 6). Another reasonably common Doppler presentation is the retrograde (upside down) waveform (Fig 6), which represents reverse flow (blood moving in the opposite direction). In the foot, this can be a reconstitution from a more distal artery such as a communicating artery of the peroneal or the plantar arch. It can also be due to a collateral vessel as a result of a proximal arterial occlusion.

### Irregular waveforms

Doppler signals are pulsatile (i.e. cycle with each heart beat) [25], therefore presence of an irregular Doppler waveform is also particularly important to highlight, as an undiagnosed arrythmia is potentially life threatening. Podiatrists working with atrial fibrillation clinical networks have shown that reporting of irregular foot pulses resulted in detection and subsequent treatment of life-threatening atrial fibrillation [26]. This has resulted in the development of standard podiatry pathways for atrial fibrillation [27] with scope for this proactive approach to be adopted by all podiatrists.

## Conclusion

Handheld Doppler is one of the most important and frequently used vascular assessment tools currently available to podiatrists. Used in combination with clinical history, signs, symptoms, pulse palpation and pressure measurement, podiatrists use Doppler to detect PAD at the earliest opportunity and make subsequent care plans, involving general practitioners, exercise physiologists and vascular surgery teams. These care plans serve to not only saves limbs through early detection and management of PAD and associated wounds, but to also save or improve lives through ensuring appropriate proactive cardiovascular risk management.

## Data Availability

Not applicable.
